# Metagenomic sequencing in encephalitis diagnostics: Challenges and opportunities in clinical settings

**DOI:** 10.1371/journal.pone.0358189

**Published:** 2026-09-15

**Authors:** Erika Stentoft, Josefin Olausson, Emilio Rudbeck, Marie Studahl, Hedvig E. Jakobsson

**Affiliations:** 1 Department of Infectious Diseases, Sahlgrenska University Hospital, Region Västra Götaland, Gothenburg, Sweden; 2 Department of Infectious Diseases, Institute of Biomedicine, Sahlgrenska Academy, University of Gothenburg, Gothenburg, Sweden; 3 Department of Clinical Microbiology, Sahlgrenska University Hospital, Region Västra Götaland, Gothenburg, Sweden; 4 Bioinformatics and Data Centre, University of Gothenburg, Gothenburg, Sweden; Cleveland Clinic Abu Dhabi, UNITED ARAB EMIRATES

## Abstract

The primary aim of this study was to determine whether metagenomic next-generation sequencing (mNGS) can identify potential microbial agents responsible for encephalitis of unknown origin in immunocompetent patients, thereby enhancing clinical diagnostics. Cerebrospinal fluid samples from well-characterized patients (n = 17) diagnosed with encephalitis of unknown origin, according to Swedish national guidelines, were sequenced using mNGS using the Ion Torrent platform and analyzed using bioinformatic platforms. Samples from patients with known viral CNS infections i.e. HSV-2 meningitis (n = 4), VZV CNS infections (n = 3), enterovirus meningitis (n = 2), JCV CNS infection (n = 2) were used as controls for the methodology (n = 11). No viral agents were detected in 16/17 CSF samples from patients with encephalitis of unknown etiology. 13/17 CSF samples were analysed for the most common autoimmune antibodies and were negative. In one CSF sample from patients with encephalitis of unknown origin a Human pegivirus (HPgV) was detected. In 9/11 control CSF samples from patients with CNS infections, RNA or DNA of the known virus were detected. The main conclusion in this study was that the negative results were related to that the majority of included patients were immunocompetent. The finding of HPgV in a patient with unknown encephalitis was judged as a bystander. However, mNGS might detect more pathogens in other patient cohorts and this study implicates that a close collaboration between the clinical laboratory and the clinicians enables a safe implementation of metagenomics.

## Introduction

Infectious encephalitis is a rare condition with an incidence of approximately 5/100 000 [1], but associated with a high risk of mortality and neurological and neurocognitive sequels at long-term follow-up [2]. The majority of microbiologically verified infections of the central nervous system (CNS) manifested as encephalitis are of viral origin while bacterial, fungal and protozoal infections are more rarely diagnosed. The three most common causes of viral encephalitis in Sweden are tick-borne encephalitis virus (TBEV), varicella zoster virus (VZV) and herpes simplex virus 1 (HSV-1) [3]. The occurrence of TBEV differ within the country due to geographical variations in epidemiology [4]. Despite advances in molecular diagnostics, such as RT-PCR and multiplex PCR panels, the microbiological cause is still unknown in approximately 50% of suspected viral central nervous system (CNS) infections [5,6]. Furthermore, in several other CNS diseases of autoimmune, autoinflammatory or malignant origin, the patient may present with symptoms similar to a CNS infection and subsequently there is a need of distinguishing these entities diagnostically [7]. Other treatments than antimicrobial therapy might be needed, e.g., immunosuppressive drugs such as steroids, which may worsen an undetected infection and thereby be potentially hazardous for the patient. Further investigation regarding diagnostic opportunities and possibility to find a differential diagnose is therefore essential. Metagenomic next generation sequencing (mNGS) of cerebrospinal fluid (CSF) has emerged as an auspicious method to identify an extensive range of pathogens, both previously known encephalitis agents and novel organisms. In a single test sequencing [8,9] mNGS unconditionally sequence all genetic material in a sample and millions of sequences are thereafter analyzed by bioinformatics. This technique differs from previous hypothesis-driven targeted microbial methods to detect pathogens. An increasing number of reports are published, describing the usefulness of the technique in diagnosing previously unknown infections of the central nervous system by mNGS using CSF or brain tissue [10,11]. The primary objective of this study was to determine whether metagenomic next-generation sequencing (mNGS) can identify potential microbial pathogens responsible for encephalitis of unknown origin in immunocompetent patients, thereby advancing diagnostic approaches.

## Materials and methods

### Study design, study participants, sample selection and etiological analyses

The study complied with the Declaration of Helsinki and was approved by the Regional Ethics Review Board in Gothenburg (191−18). Due to the retrospective nature of the study, the Regional Ethics Review Board in Gothenburg waived the need of obtaining informed consent.

This retrospective study collected CSF samples from patients with encephalitis of unknown origin for analysis with mNGS. The medical records of patients aged >16 years of age admitted to the Department of Infectious Diseases, Sahlgrenska University Hospital with a confirmed or suspected viral CNS infection between 2011–2018 were reviewed 2019-10-14, with follow-up 2025-05-16. A case of encephalitis was defined as follows according to the International Encephalitis Consortium: an altered mental status (decreased or altered consciousness, lethargy or behavior disorder) and presence of symptoms for >24 hours. Furthermore, at least two of the following criteria had to be fulfilled; fever ≥38°C (documented within 72 hours before or after onset of neurological symptoms), generalized or partial seizures (in patients without pre-existing epileptic disorder), recent onset of focal neurological symptoms, a CSF cell count ≥5 leukocytes/mm^3^, imaging of the brain parenchyma suggesting encephalitis, and EEG abnormality consistent with encephalitis, and not attributed to another disease [12].

**Fig 1:**
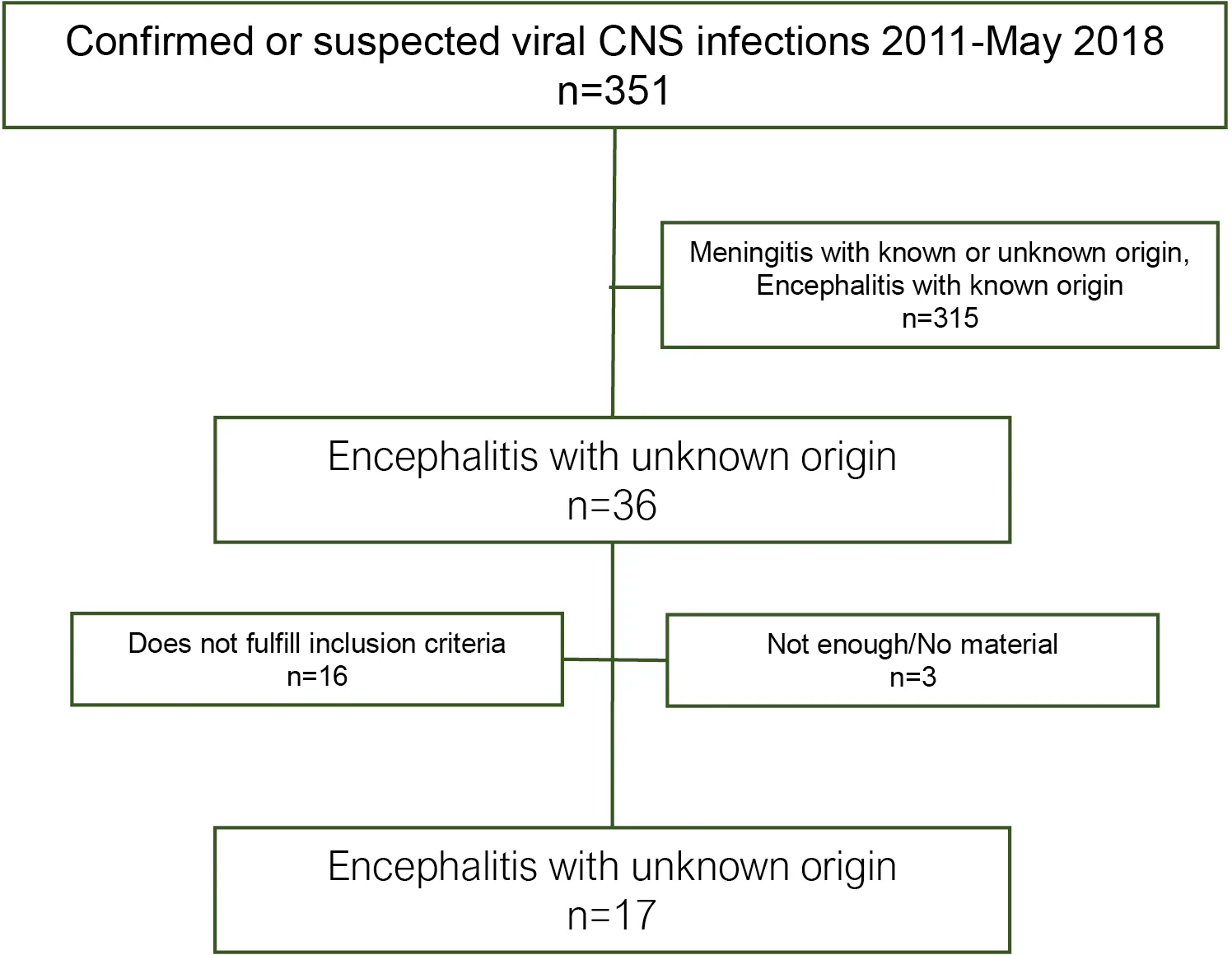
Patient inclusion. Flow-chart presenting included and analyzed CSF patient samples with unknown origin of encephalitis.

CSF samples of patients with encephalitis were drawn at admission to hospital and etiological investigation in accordance to Swedish national guidelines [13]. Analysis of CSF samples included bacteriological culture, quantitative PCR specific for HSV-1, HSV-2, VZV, EV, CMV and HHV-6 [14]. The etiological testing also included serology for TBEV in serum (ECDC definition) [15]. Encephalitis cases negative for the mentioned analyses who fulfilled the inclusion criteria of encephalitis and where a stored CSF sample was available were included in the study as encephalitis of unknown origin. The flow chart of included patients in the study is in Fig 1. Retrospective analysis of autoimmune antibodies in CSF was performed for GABA-B, DPPX, LGI1, AMPAR1/2, CASPR2, GAD and NMDAR at the Clinical Immunology, Sahlgrenska university hospital [16].

The control group was recruited from the Virology Laboratory and consisted of 11 patients with meningitis or encephalitis with an identified viral pathogen (4 samples from HSV-2 meningitis, 3 samples from VZV CNS infections, 2 samples from EV meningitis, and 2 samples from JC-virus (JCV)) CNS infections between 2014−2018. All CSF samples were stored at −20 °C.

A clinical judgement of positive CSF mNGS results as a cause of disease in samples from encephalitis of unknown origin was performed by two of the authors (E.S and M.S).

### Nucleic acid extraction from cerebrospinal fluid samples

Total nucleic acid was extracted from 400 µl of cerebrospinal fluid using the MagNAPure Compact Nucleic Acid Isolation Kit I (Roche Diagnostics, Indianapolis, IN, USA) on the MagNAPure compact automated extractor. RNA and DNA concentrations were assessed using the Qubit 4.0 Fluorometer (Thermo Fisher Scientific, Waltham, MA, USA) using the dsDNA HS Assay Kit and RNA HS assay kit (Thermo Fisher). Even if nucleic acid concentrations were too low to assess, library preparation was performed. The cell line P3HR1 (HTB-62, American Type Culture Collection, ATCC, USA), transfected with Epstein Barr virus, were used as combined negative and positive control, showing both stability in viral reads, as well as human and laboratory background. Control samples were processed in parallel with the patient samples during all the laboratory steps.

### Library preparation and next generation sequencing

Each extraction were divided into two samples, where DNA and RNA libraries were prepared. DNA libraries were prepared as previously described [17]. Briefly, using the Ion Xpress Plus Fragment Library Kit (Thermo Fisher) on the AB Library Builder System (Thermo Fisher), samples were fragmented and barcoded. RNA libraries where prepared using Ion Total RNA-Seq Kit v2 (ThermoFisher) on the AB Library Builder System (ThermoFisher). Every RNA sample was divided into two fractions, fragmented with two different times per sample (1 min and 3 min). RNA and DNA libraries were amplified, selecting the number of amplification cycles according to the sample input concentration, varying between 10–27 cycles, where amplification was controlled using the High Sensitivity D1000 DNA Kit on Agilent 2200 TapeStation system (Agilent Technologies) every third cycle from 10 cycles (DNA) or 14 cycles (RNA) and up, to ensure that primer dimers and concatamers did not become abundant. Size selection was performed using the Pippin Prep platform (Sage Science, Beverly, MA, USA) with 2% dye free agarose gel cassette, with range 120–320 bp. Subsequently, libraries were purified using Agencourt AMPure XP (Beckman Coulter, Brea, CA, USA). Finally, libraries were quantified using qPCR with the Ion Library TaqMan Quantitation Kit (Thermo Fisher) and the size estimated using High Sensitivity D1000 DNA Kit on Agilent 2200 TapeStation system (Agilent Technologies). For template preparation, libraries were pooled to a final concentration of 50 pM, if obtainable. For libraries with concentration lower than 50 pM, libraries were pooled to the available concentration. Thereafter, the Ion Chef Platform was used to ligate the libraries onto spheres using the Ion 540 Kit-Chef (Thermo Fisher). Following clonal amplification, libraries were loaded onto Ion 540 Chip and sequencing was performed on the S5 System (Prime; Thermo Fisher) according to the manufacturer’s protocol for 200 bp read length.

### Bioinformatic analysis

The bioinformatic pipeline Pathogen detection for Research and Clinical Applications (PaRCA), were used for all bioinformatic classification, as previously described [17]. RNA reads were assembled using Megahit (1.1.3) using a minimum contig length of 200 and the RNA setting. After assembly, the dataset was mapped to the contigs using BBMap with a k-mer filter of 22, a maximum indel of 80 and perfect mode. Mapped reads were individually counted and tagged and results saved for future use within the pipeline.

#### Additional bioinformatic analysis.

Where viral results were inconclusive or unexpected, we sought to find alternative ways to determine the presence of the HPgV. The samples were first assessed for quality using FastQC, followed by trimming using fastp, and assessed again post-trimming with FastQC https://qubeshub.org/resources/fastqc. Trimming was done using the standard settings in fastp, meaning that the reads were trimmed based on length, undetermined bases, and bases of too low quality [18]. The trimmed reads were then assembled into scaffolds with SPAdes, using the inbuilt Ion Torrent and rnaviral modes [19]. Once this was done the resulting fasta-files were analyzed for completeness using CheckV v1.0.1 https://bitbucket.org/berkeleylab/checkv which searches each assembled sequence against a virus gene database [20]. Through this, the sequences are assessed and a value on genome completeness is received. The final step in the analysis was to determine the taxonomy of each sequence. This was done manually with the megablast available at https://blast.ncbi.nlm.nih.gov where each sequence was taxonomically assigned based on its top hit. Select output data was then pasted into a table and presented via CLC along with each sample’s assembly.

#### Calculations and statistical analysis.

A ratio between sample rpm (reads per total million) and control rpm were calculated, where a ratio ≤ 10 was considered a contamination.

The following formula was used to calculate for theoretical JCV reads:


$$\begin{array}{c} Pathogen\;read=\frac{L}{\left(\frac{C_{H}\;\times \;G_{H}\times V}{C_{P}\;\times \;G_{P}}\times \;M^{-\text{1}}\right)} \end{array}$$

Pathogen genome size (G_P_), the diploid human genome size of 6.5 billion base pairs (G_H_), pathogen copy according to PCR per milliliter (C_P_), whole blood cell count per milliliter (C_H_), and adjusted according to the volume (V), sequencing library size (L) and mappability in percent (M) to remove major contaminants [17].

Pathogen reads classified with PaRCA were uploaded to the CLC genomics workbench (Ver. 22, Qiagen) to perform and plot coverage analyzis with the inbuilt tool “Map to reference”. Read distribution over the reference genomes was evaluated with total number of pathogen reads in mind. Reference genomes used were NC_001798.2 (HSV2), NC_001348 (VZV), NC_00196 (JCV), NC_007605 (EBV), NC_001472 (EV), NC_001710.1 (HPgV).

## Results

### mNGS in patients with encephalitis of unknown origin

17 patients met the criteria for inclusion of encephalitis of unknown origin: both fulfilling encephalitis criteria, negative basic etiological analyzes and had sufficient amount of CSF sample stored in −20 °C (Fig 1). A summary of the clinical characteristics of the patients included is presented in Table 1. CSF samples were negative regarding bacterial culture, and also PCR specific targeting VZV, HSV-1, HSV-2, EV (enterovirus), human herpes virus 6 (HHV-6), cytomegalovirus (CMV) and Epstein-Barr virus (EBV). TBE serology in serum was negative.

**Table 1:** Characteristics of included patients.

Total number, *n*	17
Age, median, range (years)	38 (21–84)
Male sex, n (%)	14 (82)
Time of hospitalization, median, range (days)	10 (4–54)
**Co-Morbidity:**	
Malignancy or previous malignancy*, *n*	3
Psychiatric disorder, *n*	2
Neurological disease, *n*	1
Hypertension, *n*	1
**CSF (initial lp):**	
Pleocytosis, *n* (%)	17 (100)
Sp-Monocytes (x10^6^/l) median, (range)	11 (0–36)
Sp-Lymphocytes (x10^6^/l) median, (range)	100 (0–341)
Sp-Neutrophils (x10^6^/l) median, (range)	0 (0–164)
Sp-Erythrocytes (x10^6^/l) median, (range)	0 (0–136)
Sp-Albumin (mg/l) median, (range)	588.5 (202–1330) 1 nd
Sp-Lactate (mmol/l median, (range)	3 (1.5–4.7) 2 nd
CT, *n* (%) of which pathological, *n*	12 (71), 1
MRI, *n* (%) of which pathological, *n*	13 (76), 2
EEG, *n* (%) of which pathological, *n*	12 (71), 9
Prodromal symptoms, *n* (%)	8 (47)
**Clinical features: *Signs and symptoms***	
Fever, *n* (%)	16 (94)
Confusion, *n* (%)	14 (82)
Dysphasia, *n* (%)	4 (24)
Dysarthria, *n* (%)	3 (18)
Altered consciousness, *n* (%)	2 (12)
Paresis in extremities, *n* (%)	2 (12)
Nystagmus, *n* (%)	2 (12)
Impaired balance, *n* (%)	2 (12)
Cranial nerve palsy, *n* (%)	1 (6)
Double vision, *n* (%)	1 (6)
Loss of sensation, *n* (%)	1 (6)
Ataxia, *n* (%)	1 (6)
Epileptic seizure, *n* (%)	1 (6)

Characteristics of the patients with encephalitis of unknown origin and their signs and symptoms during the initial week of illness, laboratory findings and result of examinations. CT: Computed tomography; MRI: Magnetic Resonance imaging; EEG: Electroencephalogram; lp: Lumbar puncture; sp: spinal. * T-cell lymphoma, previous prostate cancer, previous solitary malignant plasmocytoma.

Furthermore, the majority of the CSF samples were analyzed for the most common autoimmune antibodies in encephalitis at the Department of Clinical Immunology, Sahlgrenska university hospital (patient no. 7, 10, 16 and 17 lacked enough material and were excluded from the analysis). All analyzed samples were negative for antibodies against Gamma-aminobutyric acid-B (GABA-B), Dipetidyl-peptidase-like protein 6 (DPPX2), Leucine-rich glioma inactivated-1 (LGI1), α-amino-3-hydroxy-5-methyl-4-isoxazolepropionic acid receptor (AMPAR1/2), Anti-contactin-associated protein-like 2 (CASPR2), Glutamin acid decarboxylase (GAD) and N-methyl-D-aspartate receptor (NMDAR).

The sequencing results of the 17 patients are presented in Table 2. No viral agents were detected in 16/17 CSF samples from patients with encephalitis of unknown etiology. However, human pegivirus (HPgV) was found in three patient samples (Patient no. 10, 16, and 17; S1 Table) by the classifier PaRCA.

**Table 2:** Overview of sequencing results of CSF samples from the patients with unknown encephalitis.

StudyID	DNA/RNA	Total IT reads	Classified Reads	Human (Reads)	Viral (Reads)	Found Pathogen (RPM)
no. 1	DNA	5 040 330	4 326 889	4 309 908	1 376	
	RNA	2 939 628	1 137 640	850 128	663	
no. 2	DNA	4 843 337	4 102 557	4 085 895	1 329	No reads EBV (1)
	RNA	10 240 009	7 118 613	6 031 294	855	
no. 3	DNA	7 309 297	6 245 946	6 223 632	2 158	
	RNA	34 679 691	32 766 549	23 389 956	25 132	
no. 4	DNA	1 747 406	1 586 510	1 579 287	631	
	RNA	40 373 737	36 922 180	26 634 284	37 398	
no. 5	DNA	12 483 187	11 005 305	10 959 148	3 278	
	RNA	4 600 913	4 150 212	3 531 001	27	
no. 6	DNA	6 903 288	6 193 548	6 169 679	1 859	
	RNA	19 776 643	17 981 926	15 963 020	18 260	
no. 7	DNA	5 146 444	4 709 665	4 691 423	1 442	
	RNA	30 462 562	26 743 993	22 896 148	7 266	
no. 8	DNA	5 883 710	5 544 419	5 524 932	1 704	
	RNA	Tech. Fail				
no. 9	DNA	23 732 991	21 881 940	21 796 635	6 156	
	RNA	16 440 417	13 624 089	6 279 320	2 152	
no. 10	DNA	15 737 469	13 916 756	13 861 252	4 374	
	RNA	7 146 098	1 584 005	954 656	5 413	
no. 11	DNA	15 216 012	13 572 267	13 491 921	5 247	
	RNA	6 293 190	2 931 051	2 639 828	4 298	
no. 12	DNA	2 299 228	2 118 719	2 114 115	915	No reads EBV (1)
	RNA	52 152 989	42 451 577	41 462 625	68 459	
no. 13	DNA	14 604 551	12 497 095	12 445 506	3 684	
	RNA	2 767 188	602 104	348 369	27	
no. 14	DNA	3 590 052	3 247 399	3 236 062	1 025	
	RNA	3 114 548	1 087 980	584 657	497	
no. 15	DNA	38 737 623	35 773 470	35 635 172	11 118	
	RNA	18 181 088	16 594 028	14 700 139	6 320	
no. 16	DNA	8 058 262	7 516 666	7 487 547	3 319	No reads Pegivirus C (7 823)
	RNA	69 583 204	62 380 562	58 637 149	1 091 630	
no. 17	DNA	14 362 931	13 222 961	13 026 044	4 145	
	RNA	5 256 647	3 603 417	587 560	9 308	

The table presents type of sample analyzed, total number of reads, classified reads, human versus viral reads and if any pathogen was found by mNGS. EBV: Epstein-Barr Virus RPM: Reads per million.

**Fig 2:**
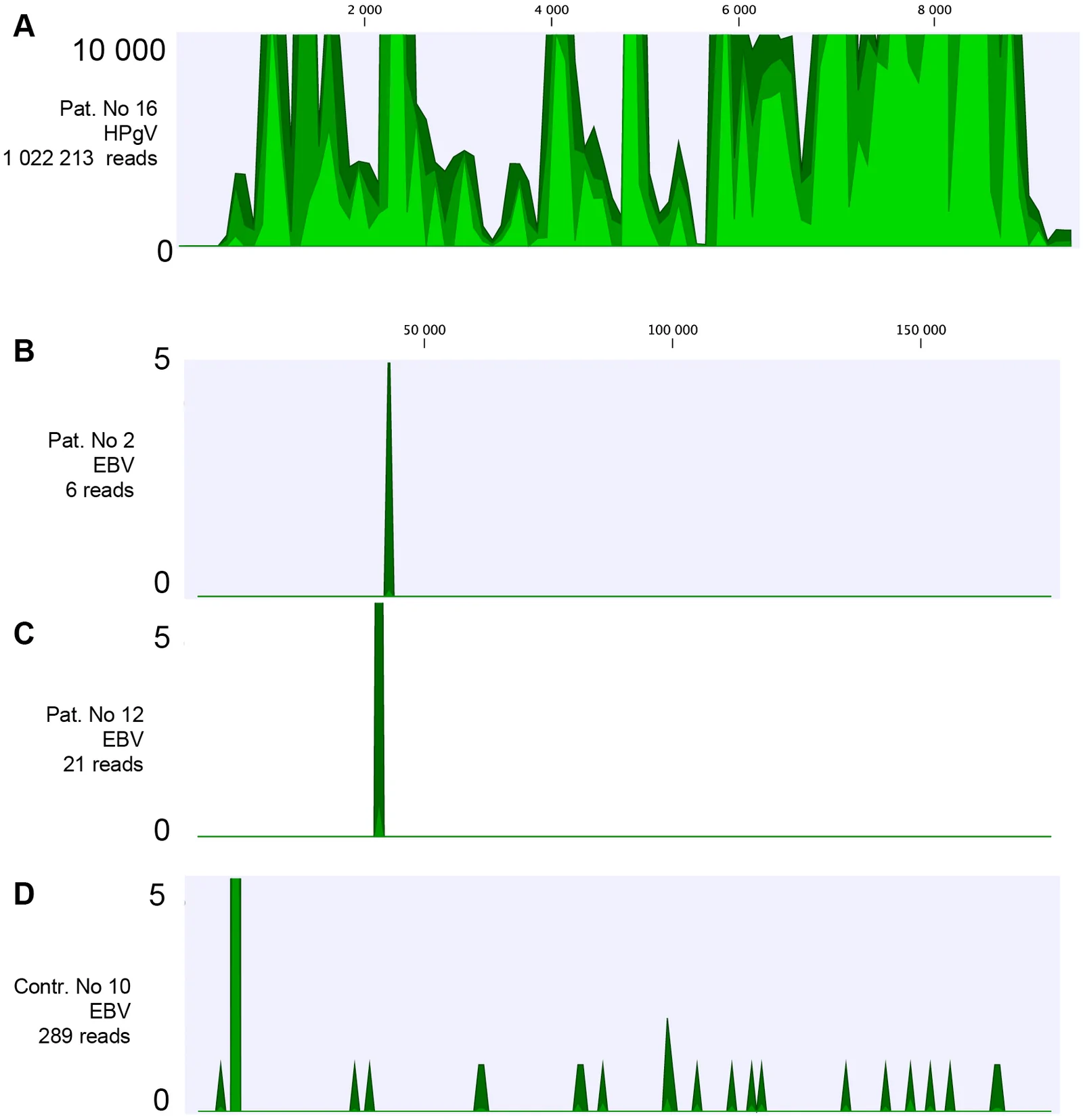
Genome alignment of viral findings. Coverage density plot of sequencing reads from respective sample and control detected in PaRCA aligned to reference genomes of Pegivirus C (A-C) and Epstein Barr virus (D) and Number of reads (y-axis) at each nucleotide position of the genome (x-axis) depicted in green. Dark green represents peak, bright green average, and light green minimum coverage for respective sections of the genome. A depth cut-off was applied at 10 000 for A, and 5 for B-D and to accurately visualize the coverage.

In order to further investigate the samples positive for HPgV an alternative assembly bioinformatic method was performed (see Methods under Additional bioinformatic analysis) to validate the results from the classifier. When applying this method patient no. 17 was excluded due to failed assembly, however patient no. 10 and patient no. 16 contained enough pegiviral sequences to obtain a result in detectable scaffolds. Further, following assessment in the software CheckV only patient no. 16 qualified with enough sequences aligned to Pegivirus. Once the analysis with CheckV had been used to assess the patient, it was also found that the reads from this patient mapped well onto a reference genome. (Fig 2A).

Minute levels of EBV was found with RNA sequencing in CSF samples from patient no. 2 and patient no. 12 (Table 2). However, coverage analyzes showed that all reads corresponded to the same sequence around 40’000 bp into the genome, indicating an amplification error and false positive results (Fig 2B and C). In one of the CSF samples, no. 8, the RNA sequencing failed.

The majority of the patients (16/17) were immunocompetent, whereas one 72-year old patient (patient no. 14) was judged as immunosuppressed due to a T-cell lymphoma. Further, two patients (patient no. 2 and 9) had a malignancy in their previous medical history; A 67-year old patient had surgery for prostate cancer more than 10 years ago, and one 85-year old patient had received treatment with radiotherapy against a malignant plasmocytoma in the hip bone and was declared cancer free.

All patients, except one (patient no. 4), were concluded to have an episodical onset of neurological symptoms and no chronic illness. Patient no. 4 was transferred to the Department of Neurology on suspicion of encephalitis of autoimmune/inflammatory origin. The investigation was negative, including autoimmune antibodies in CSF, and the patient was treated with corticosteroids for six months. The patient recovered but had some remaining problems with short-term memory and no conclusive diagnosis was established. The patient was followed during several years, however with no signs of relapse.

HPgV was detected in cerebrospinal fluid (CSF) from a 38-year-old previously healthy man who presented with clinical signs of meningoencephalitis, including mononuclear pleocytosis (142 x10⁶/L), EEG abnormalities in the right frontotemporal region. He required initial intensive care due to severe confusion. Brain imaging (CT and MRI) was unremarkable, and extensive microbiological testing, including PCR and serology for common neurotropic pathogens, was negative. Rapid improvement occurred following four days of intravenous acyclovir and led to discharge. He was readmitted one week later with headache and paresthesia in all extremities. CSF cell count had decreased (44 x10⁶/L), and repeated microbiological tests remained negative. EEG showed persistent low-frequency activity in the temporal lobes. No antiviral treatment was given during the second admission, and the symptoms were attributed to a stress reaction. At follow-up, the patient reported residual headache but had otherwise recovered; Mini-Mental Test score was 29/30.

### mNGS analysis of control patients

The sequencing results in the CSF samples from the control patients are shown in Table 3 and mNGS confirmed previous quantitative PCR results for nine out of eleven known pathogens of the control patients.

**Table 3:** Overview of sequencing results in the CSF samples from the control patients.

StudyID	Expected find	Log	DNA/RNA	Total IT reads	Classified Reads	Human (Reads)	Viral (Reads)	Found Pathogen (RPM)
no. 1	HSV-2	1.7	DNA	988 272	877 180	874 344	304	HSV-2 (63) HSV-2 (488)
			RNA	8 579 859	6 748 366	5 677 971	11 720	
no. 2	JCV	2.06	DNA	9 335 522	8 034 422	7 999 656	2 960	negative
			RNA	4 364 079	3 543 345	554 481	775	
no. 3	VZV	4.33	DNA	2 991 429	2 578 428	2 569 838	928	VZV (13) VZV (56)
			RNA	34 838 228	31 892 108	28 539 068	7 903	
no. 4	HSV-2	4.02	DNA	8 122 942	7 397 777	7 374 080	2 334	No reads HSV-2 (28)
			RNA	35 961 036	31 694 999	27 861 955	17 298	
no. 5	EV	4.38	DNA	10 473 829	9 373 460	9 341 165	2 894	No reads EV (155)
			RNA	9 174 275	8 186 512	7 579 165	8 194	
no. 6	VZV	4.44	DNA	6 036 585	5 474 706	5 454 046	2 603	VZV (189) VZV (653)
			RNA	8 646 037	6 931 635	5 486 885	3 080	
no. 7	EV	4.3	DNA	21 485 520	19 186 447	19 124 048	6 042	No reads EV (515)
			RNA	13 300 586	12 121 497	9 713 980	11 514	
no. 8	HSV-2	3.43	DNA	15 332 276	14 174 891	14 127 043	4 276	HSV-2 (1) HSV-2 (105)
			RNA	18 346 002	16 021 546	12 484 496	23 028	
no. 9	JCV	3.8	DNA	12 490 492	11 307 869	11 222 064	3 654	negative
			RNA	2 276 814	387 475	133 617	153	
no. 10	VZV	2.28	DNA	5 558 737	4 998 549	4 983 048	1 906	VZV (24), EBV (4) VZV (193), EBV (120)
			RNA	3 404 218	2 906 505	2 629 492	2 646	
no. 11	HSV-2	4.32	DNA	14 882 259	13 811 171	13 747 370	4 729	HSV-2 (29) No reads
			RNA	9 650 648	8 880 881	6 877 533	5 451	

The table presents control samples and expected finding as defined by quantitative-PCR, logaritmic value, total number of reads, classified reads, human versus viral reads and results from mNGS. Log: Logarithmic value of genome equivalents/mL. HSV-2: Herpes simplex virus 2, VZV: Varicella Zoster virus, JCV: JC polyomavirus, EV: Enterovirus, EBV: Epstein-Barr virus. RPM: Reads per million.

Reads corresponding to the viral pathogen VZV (n = 3) were detected using both DNA and RNA sequencing in all samples. EV (n = 2) was, as expected, only detected using RNA sequencing in the two samples.

In the four control patients with HSV-2, both DNA and RNA reads were detected by sequencing in two of the samples, while in one sample it was only detected using RNA sequencing and in the last sample, HSV-2 was only found using DNA sequencing. JCV (n = 2) was detected neither in DNA nor in RNA sequencing.

In one CSF sample, from a control patient with VZV CNS infection, both VZV and EBV were found with mNGS. EBV was not detected with previously performed routine diagnostics with quantitative PCR, however, mapping of the reads against the reference genome showed that the reads were distributed evenly over the genome, indicating a correct finding (Fig 2D).

### mNGS analysis of cell line controls

Cell line controls were simultaneously sequenced with the patient samples in order to detect contaminations and evaluate the sequencing performance. Cell line controls originates from P3HR1 which is an human cell line with EBV incorporated into the genome. The DNA sequencing showed high reproducibilty, while the RNA sequencing had higher deviations (S1 Fig). Cell line controls were used to subtract reads from the patient samples that corresponds to contaminations (S1 Table).

## Discussion

In this single center retrospective study of immunocompetent patients with encephalitis of unknown origin, mNGS of CSF samples did not add etiological yield. The patients fulfilled international criteria of encephalitis of unknown origin, and analyses for the most common and relevant viral agents and autoimmune antibodies associated with encephalitis were negative. When applying mNGS in this cohort, no clinically relevant pathogens were detected. In one patient, HPgV was detected with mNGS and judged as true positive following bioinformatic analysis, however, no confirming PCR was performed, which would be necessary in order to decisively verify the results. Several studies have published findings of HPgV, detected either by PCR or metagenomic sequencing, in different specimens such as CSF, serum [21,22] or bone biopsy [23]. It has been regarded as a non-pathogenic lymphotropic virus, and has hitherto not been causally linked to any clinical human disease. HPgV might be looked upon as an innocent bystander, especially during CNS coinfections, illustrated by the case published by Liu et al with AIDS and encephalitis, caused by toxoplasmosis and fungal infection [24]. However, in encephalitis patients the significance of a finding of HPgV RNA where no other pathogens are detected has been discussed [21,25]. A damaged blood-brain-barrier during encephalitis could facilitate a passive transportation of HPgV since viremia may last several years after infection. Another plausible source of contamination of blood into the CSF sample is the risk of a minor bleeding during lumbar puncture, however, this was not the case with the CSF sample from the patient in this study where HPgV was detected. The lack of evidence of HPgV as an encephalitic virus lead us to interpret the finding of the virus as a probable bystander of unknown significance.

Similarly, one of the control patients, namely the patient with VZV CNS infection, had small amounts of EBV in CSF, detected with mNGS with both DNA and RNA sequencing. This finding, while true, was interpreted without clinical significance. Since EBV primarily resides in B lymphocytes, EBV DNA in CSF may simply reflect human cells infiltrating the CNS during inflammation, although a low-grade viral replication cannot be ruled out. CSF EBV DNA detection by PCR as a possible bystander to other infectious CNS diseases has been recognized since the era of PCR started [26–28]. Recently a retrospective study of 807 patients, 1005 CSF samples were analyzed by specific PCR and 74 (7.4%) tested positive for EBV DNA. The majority of patients who tested positive for EBV DNA had neuroinflammatory conditions with discernible pathogens, a few patients had malignancies and among the patients with no possible etiology other than EBV, serum EBV VCA IgM was detected only in one patient indicating that EBV DNA detection in CSF very seldom is associated with encephalitis caused by EBV [29]. However, the discussion about detection of viral sequences which are referred to as “bystanders”, such as EBV and HPgV, might be nuanced in future studies. A recent publication found an increased 10 week mortality in HIV-negative patients with cryptococcal meningitis and EBV DNA detected in CSF by mNGS compared to patients without EBV DNA in CSF [30].

Patients with encephalitis of unknown origin are a diagnostic challenge. The patients in this study have undergone multiple analyses, such as standard microbiology, and retrospectively analyses of CSF with mNGS and autoimmune antibodies which were found negative. Although patients were negative for the most commonly tested autoimmune antibodies in the acute stage, it is still a possibility with autoimmunity development in a later phase. However, the medical records were scrutinized, and no relapse or development of any other chronic neurological disease were noticed. Negative result by mNGS can not exclude viral CNS infections, since in some infections, viral RNA might not be detected in the CSF although the brain is infected, which is the case in subacute sclerosing panencephalitis (SSPE) caused by measles virus and flaviviruses such as tick-borne encephalitis virus (TBEV) [3,31].

The quality and outcome of mNGS depend on several technical aspects causing possible bias and must be considered [32].

The method was capable of detecting all but one of the pathogens (JCV) from the control patients, independent of viral load in the samples. The samples with JCV showed no pleocytosis, had a normal protein and glucose content. When applying the calculation for theoretical pathogen reads previously published [17], control no. 2 had 0.6 theoretical reads, while control no. 9 had 49 theoretical reads. The negative results of control no. 2 could therefore be explained by a lack of depth in the sequencing. This could not explain patient no. 9, however this patient had high levels of erythrocytes which might have masked the results, and is not accounted for in the calculation (S2 Table). Repeat PCR excluded viral degradation since samples were still positive for JCV. In patients with progressive multifocal leukoencephalopathy (PML) standard clinical CSF parameters with normal levels of leukocytes are prevalent [33] and other studies have indicated that there might be a higher likelihood of sequence detection in mNGS in CSF samples with higher number of leukocytes, high protein and low glucose [34]. Thus, clinically it might be a higher probability of detecting viral pathogens in patients with marked CSF abnormalities, even though mNGS as a technique loses sensitivity with a higher human background.

Previous studies indicate that low yield of nucleic acid is a challenge to create high quality libraries [35]. Data from this study with samples containing low amounts of enterovirus as well as RNA of several other viruses in the control patients still generated high quality sequencing data indicating high sensitivity despite DNA or RNA viral loads. This was likely due to that all RNA samples were run in duplicate which gained enough sequencing data when combining them. However, it is a possibility that the results from sequencing might be false negative, especially where no viral reads were detected in one of the duplicates. It is known that RNA in CSF is easily degraded. In our case, the samples were not snap-frozen at sampling point and were further stored in minus twenty degrees for an extensive time before sequencing which could have influenced the result [36,37].

Environmental contamination of reagents, equipment or the laboratory workspace, as well as choice of bioinformatic pipeline are all of critical importance when interpreting sequencing results, and must be considered [38–40]. RNA sequencing inherently poses additional issues compared to DNA sequencing, the potential of high sensitivity also bring higher complexity of reagent, laboratory and bioinformatic contaminations. Furthermore, the low RNA input might lead to amplification bias, and short sequence lengths to sequencing bias. Together, this might mask relevant results [41]. The cell line control used in this study efficiently removed genomic contaminants leaving only a few plausible findings to proceed with using additional post-classification analyses.

Depending on which bioinformatic pipeline used to analyze the sequencing results, the outcome might vary. Therefore it is important to choose a pipeline that uses validated and updated databases, as well as sequence distribution along the reference genome and single sequence lengths. Here, the bioinformatic classifier used indicated that HPgV was present in three patients, however, upon further investigation only one of these samples were determined to have a true viral result. Thus, it is important to confirm results using post-classification analysis as well as BLAST, as previously described [17].

The technique is still under development and the interpretation of the results still poses a lot of thorough work [42]. Despite no causative agent found in this study of mainly immunocompetent patients, other larger cohorts have shown the potential of mNGS in adding diagnostic yield and influence treatment in patients with CNS infections, especially in immunosuppressed [10,43]. Today, metagenomic sequencing is not a method for rapid diagnostics, and might be considered of more value in medical investigations where all first-line diagnostics with blood serology, intrathecal antibodies, and quantitative and multiplex PCR is negative [3,44,45]. Targeted PCR is often more sensitive than mNGS when the pathogen burden is low [46], which should be taken under consideration.

## Conclusions

NGS showed limited diagnostic yield in encephalitis of unknown origin in this cohort of predominantly immunocompetent patients. The detection of HPgV was interpreted as secondary in the absence of confirmative diagnostics, underscoring the challenges in distinguishing causative findings from bystanders. The failure to detect JCV may reflect the lower sensitivity of mNGS compared with targeted PCR assays, representing an important limitation. Rather than competing approaches, targeted PCR and mNGS should be regarded as complementary diagnostic tools, combining the high sensitivity of targeted testing with the unbiased pathogen detection capacity of mNGS. Future integration into routine diagnostics will depend on improved turnaround times and continuous close collaboration between clinicians and laboratories to ensure appropriate interpretation.

## Supporting information

S1 Fig Cell line control results. Cell line controls with EBV incorporated into the genome were sequenced together with samples in all eight sequencing runs. RNA controls showed higher divergence than DNA controls. RPM: reads per million total reads.
(PDF)

S1 Table Findings in mNGS sequencing. Sequencing reads from all samples sequenced in order of runs. Table is divided to show results from DNA, as well as long and short fragmentation time of RNA. Patient samples, Control patient samples and cell line controls are included in each of the eight sequencing runs.
(XLSX)

S2 Table Calculation of theoretical reads. Table show the calculation of theoretical reads in control patient 2 and 9.
(XLSX)
