# Supplementary material for: Metagenomic sequencing in encephalitis diagnostics: Challenges and opportunities in clinical settings
Source: PLoS One. 2026 Sep 15;21(9):e0358189. doi: 10.1371/journal.pone.0358189 (PMC13577485; doi:10.1371/journal.pone.0358189)
Supplement: Supplementary file 1 [file pone.0358189.s001.pdf]

Additional figure 1

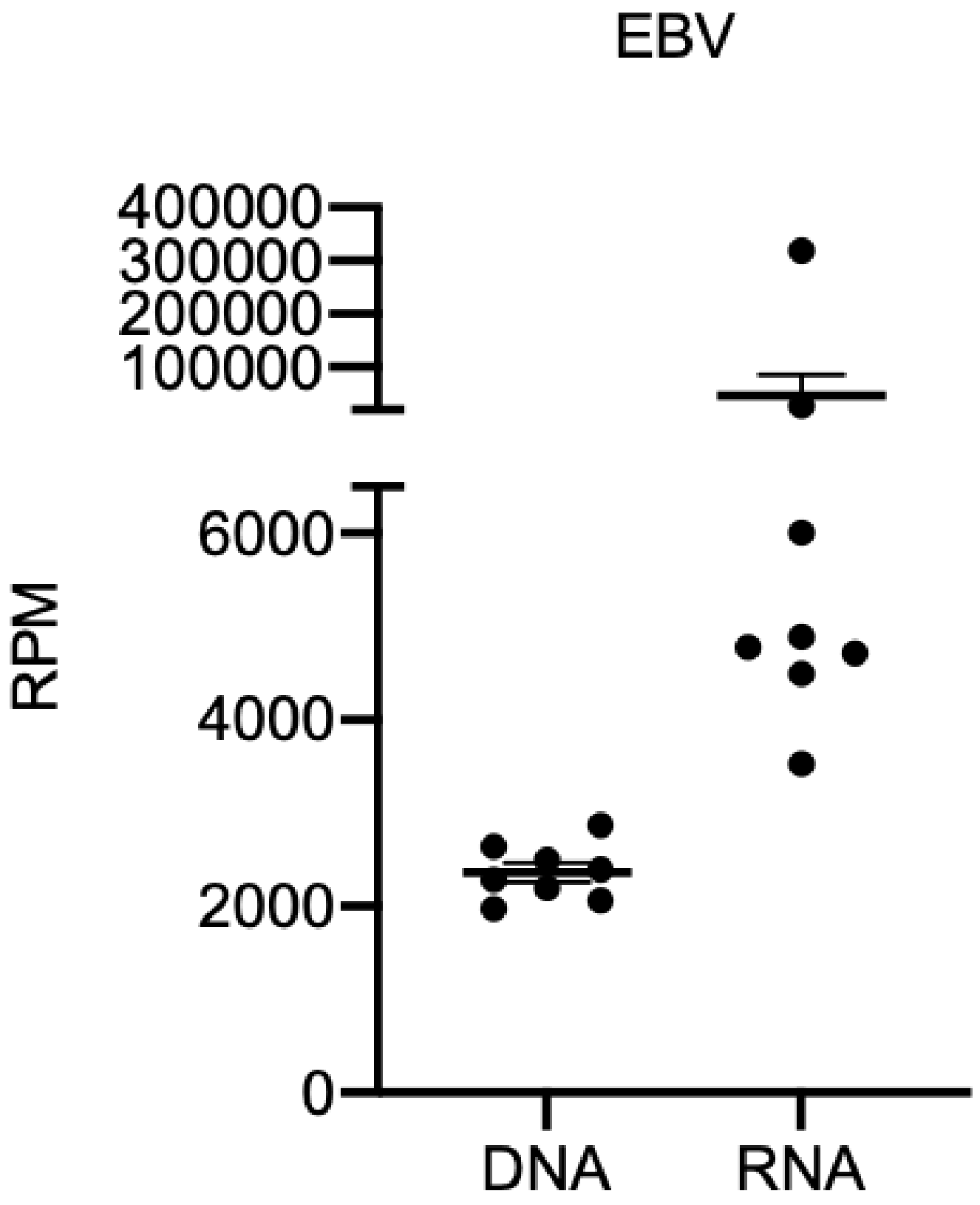

Additional figure 1. Cell line control results. Cell line controls with EBV incorporated into the genome were sequenced together with samples in all eight sequencing runs. RNA controls showed higher divergence than DNA controls. RPM: reads per million total reads.
